# Metformin suppresses adipogenesis through both AMP-activated protein kinase (AMPK)-dependent and AMPK-independent mechanisms

**DOI:** 10.1016/j.mce.2016.11.011

**Published:** 2017-01-15

**Authors:** Suet Ching Chen, Rebecca Brooks, Jessica Houskeeper, Shaun K. Bremner, Julia Dunlop, Benoit Viollet, Pamela J. Logan, Ian P. Salt, S. Faisal Ahmed, Stephen J. Yarwood

**Affiliations:** a The Developmental Endocrinology Research Group, School of Medicine, University of Glasgow, Glasgow G51 4TF, UK; b Institute of Molecular, Cell and Systems Biology, University Avenue, University of Glasgow, Glasgow G12 8QQ, UK; c Institute of Cardiovascular and Medical Sciences, University Avenue, University of Glasgow, Glasgow G12 8QQ, UK; d INSERM, U1016, Institut Cochin, Paris, France, CNRS, UMR8104, Paris, France, Université Paris Descartes, Sorbonne Paris Cité, France; e Institute of Biological Chemistry, Biophysics and Bioengineering, Edinburgh Campus, Heriot-Watt University, Edinburgh EH14 4AS, UK

**Keywords:** Adipogenesis, Osteogenesis, Metformin, Diabetes, AMPK, Mesenchymal stem cells

## Abstract

People with Type 2 diabetes mellitus (T2DM) have reduced bone mineral density and an increased risk of fractures due to altered mesenchymal stem cell (MSC) differentiation in the bone marrow. This leads to a shift in the balance of differentiation away from bone formation (osteogenesis) in favour of fat cell development (adipogenesis). The commonly used anti-diabetic drug, metformin, activates the osteogenic transcription factor Runt-related transcription factor 2 (Runx2), which may suppress adipogenesis, leading to improved bone health. Here we investigate the involvement of the metabolic enzyme, AMP-activated protein kinase (AMPK), in these protective actions of metformin. The anti-adipogenic actions of metformin were observed in multipotent C3H10T1/2 MSCs, in which metformin exerted reciprocal control over the activities of Runx2 and the adipogenic transcription factor, PPARγ, leading to suppression of adipogenesis. These effects appeared to be independent of AMPK activation but rather through the suppression of the mTOR/p70^S6K^ signalling pathway. Basal AMPK and mTOR/p70^S6K^ activity did appear to be required for adipogenesis, as demonstrated by the use of the AMPK inhibitor, compound C. This observation was further supported by using AMPK knockout mouse embryo fibroblasts (MEFs) where adipogenesis, as assessed by reduced lipid accumulation and expression of the adipogeneic transcription factor, C/EBPβ, was found to display an absolute requirement for AMPK. Further activation of AMPK in wild type MEFS, with either metformin or the AMPK-specific activator, A769662, was also associated with suppression of adipogenesis. It appears, therefore, that basal AMPK activity is required for adipogenesis and that metformin can inhibit adipogenesis through AMPK-dependent or -independent mechanisms, depending on the cellular context.

## Highlights

• Metformin can inhibit adipogenesis through AMPK-dependent or -independent mechanisms, depending on the cellular context.
• Metformin suppresses adipogenesis in C3H10T1/2 mesenchymal stem cells (MSCs), independently of AMPK.
• Metformin exerts reciprocal control over osteogenic (Runx2) and adipogenic (PPARγ) transcription factors in MSCs.
• Basal AMPK and mTOR/p70^S6K^ activity are required for adipogenesis in MSCs.

## 1 Introduction

Type 2 diabetes mellitus (T2DM) is characterized by chronic elevation of blood glucose levels because of systemic insulin resistance. In addition to the reduction of insulin sensitivity in muscle, adipose tissue and the liver, it has been noted recently that people with diabetes have increased risk of bone fractures (Janghorbani et al., 2007, Kilpadi et al., 2014, Hothersall et al., 2013). Furthermore, the use of the thiazolidinedione (TZD) antidiabetic drug class, which includes pioglitazone and rosiglitazone, has been shown to increase the risk of bone fractures and secondary osteoporosis (Lecka-Czernik, 2009, Grey et al., 2007, Schwartz et al., 2006).

TZDs act as agonists for the nuclear receptor peroxisome proliferator-activated receptor gamma (PPARγ) which is considered to be the master regulator of fat cell development (adipogenesis) (Tontonoz et al., 1994a). It is therefore thought that the detrimental effects of TZDs on bone health is through the activation of PPARγ in mesenchymal stem cells (MSCs) causing adipogenesis and, consequently, suppressing bone development (osteogenesis) (Lecka-Czernik et al., 2007, Shockley et al., 2009). Adipogenesis is regulated by a temporally induced cascade involving PPARγ and members of the CCAAT/enhancer binding protein (C/EBP) transcription factor family. In the initial stages of adipogenesis there is a transient accumulation of C/EBPβ and C/EBPδ proteins, leading to a later accumulation of C/EBPα and PPARγ (Cao et al., 1991, Yeh et al., 1995). C/EBPα and PPARγ then promote gene expression characteristic of an adipocyte phenotype and their expression remains elevated for the life of the differentiated cell (Tontonoz et al., 1994b).

Metformin has been used clinically for the treatment of T2DM since the 1960s and it is thought to function primarily through the inhibition of hepatic gluconeogenesis (Cusi et al., 1996). Metformin interferes with oxidative phosphorylation in the mitochondria by inhibiting complex I in the electron transport chain, although the exact mechanism of inhibition is not yet known (El-Mir et al., 2000, Lantier et al., 2014). Metformin has also been shown to stimulate osteogenic differentiation of MSCs towards osteoblasts *in vitro* through the trans-activation of Runt-related transcription factor 2 (Runx2), the key regulatory transcription factor for osteogenic differentiation (Jang et al., 2011) and, unlike TZDs, has been shown to be associated with a reduced risk of fractures. Osteoblast differentiation has been proposed to be dependent on the cellular energy sensor AMP-activated protein kinase (AMPK), as the expression of various osteogenic genes has been shown to be inhibited by compound C, a chemical inhibitor of AMPK, and a dominant negative form of AMPK (Banerjee et al., 1997). Furthermore, metformin stimulates AMPK activation through the inhibition of oxidative phosphorylation in hepatocytes (Zhou et al., 2001).

AMPK is a heterotrimeric serine/threonine protein kinase that acts as a cellular energy sensor due to its ability to be activated by an increase in the AMP-ATP ratio, which leads to phosphorylation of Thr172 on AMPKα by liver kinase B1 (LKB1) (Hardie, 2015, Woods et al., 2003). AMPK can also be phosphorylated and activated at Thr172 by calcium/calmodulin-dependent protein kinase kinase (CaMKK) in a Ca^2+^-dependent, AMP-independent manner (Hawley et al., 2005). AMPK functions to inhibit ATP consuming pathways and at the same time activate catabolic pathways to re-establish cellular energy homeostasis. It has also been shown that AMPK has an array of non-metabolic functions including promotion of nitric oxide synthesis and numerous anti-inflammatory actions (Jones et al., 2005, Reihill et al., 2007, Salminen et al., 2011, Morrow et al., 2003, Salt and Palmer, 2012. Recently, it has been shown that AMPK functions in cell differentiation by promoting osteogenic differentiation while suppressing adipogenic differentiation (Kanazawa et al., 2008, Vila-Bedmar et al., 2010), however, the role of AMPK in cell commitment to differentiation remains unclear. Therefore, the main aim of the current study is to determine the effect of metformin on adipogenesis and, in particular, to understand the role of the AMPK signalling pathway in these processes.

## 2 Materials and methods

### 2.1 Cell culture and induction of differentiation

AMPK α1/α2 knockout mouse embryonic fibroblasts (MEFs), C3H10T1/2 mouse mesenchymal stem cells (Clone 9; ATCC CCL-226) and 3T3-L1 preadipocytes were maintained in DMEM (41965–039, Sigma-Aldrich Ltd, Gillingham, Dorset, UK) containing 10% (v/v) FCS, 2 mM glutamine, 100 U/mL penicillin and 100 μg/ml streptomycin. To promote adipogenic differentiation, cells were cultured in the standard media supplemented with either 10 μM pioglitazone alone or in combination with 100 nM insulin, 500 μM 3-isobutyl-1-methylxanthine (IBMX) and 10 μM dexamethasone (IID medium). For osteogenic differentiation, cells were cultured in standard media supplemented with 284 μmol/L ascorbic acid, 10 mM β-glycerophosphate and 10 nM dexamethasone (AGD medium). Differentiation media was changed every 3 days.

### 2.2 Preparation of cell extracts

For the preparation of cell extracts from MEFs, the media was aspirated and then cells were washed with ice cold PBS (137 mM NaCl, 2.7 mM KCl, 10 mM Na_2_HPO_4_, 1.8 mM KH_2_PO_4_) and then either 100 μl of ice cold Triton-X100 lysis buffer (50 mM Tris-HCl pH 7.4, 50 mM NaF, 1 mM Na_4_P_2_O_7_, 1 mM EDTA, 1 mM EGTA, 250 mM mannitol, 1% (v/v) triton X-100, 0.1 mM phenylmethanesulphonylfluoride (PMSF), 0.1 mM benzamidine, 5 μg/ml soybean trypsin inhibitor, 1 mM dithiothreitol (DTT), 1 mM Na_3_VO_4_) or 1× Laemmli-sample buffer (50 mM Tris-HCl pH 6.8, 2% (w/v) SDS, 10% (v/v) glycerol, 0.1% (w/v) bromophenol blue, 50 mM DTT) was added and then cells were harvested by scraping. Lysates exracted with Triton-X100 were cleared by centrifugation (24 100 × *g,* for 5 min at 4 °C) and the supernatant stored at −20 °C. Samples lysed using 1× Laemmli-sample buffer were incubated in a sonicating water bath at 60 °C for 30 min prior to storage at −20 °C. C3H10T1/2 MSCs were harvested and nuclear extracts prepared using the Nuclear Extraction kit from Active Motif, Belgium. Briefly, the media was aspirated and cells were harvested in ice-cold PBS containing phosphatase inhibitors and centrifuged (200 × *g*, for 5 min at 4 °C) to obtain a cell pellet. The cell pellet was re-suspended in complete lysis buffer containing 10 mM DTT and phosphatase inhibitor cocktail and then centrifuged (14 000 × *g*, for 10 min at 4 °C) to obtain nuclear and supernatant fractions.

### 2.3 Western blotting

Protein samples were separated by SDS-PAGE and then transferred electrophoretically onto nitrocellulose membranes. Membranes were blocked with 5% (w/v) Marvel milk powder in TBS (20 mM Tris-HCl pH 7.5, 150 mM NaCl) for 1 h. Membranes were then incubated with primary antibodies overnight at 4 °C. Following washes with TBST (TBS supplemented with 0.5% (v/v) Tween-20), membranes were incubated with secondary antibodies for 1 h at room temperature. Antibodies were diluted to the required concentration in 50% (v/v) Sea Block (Thermo Scientific) and 50% (v/v) TBST. The primary antibodies included those purchased from Cell Signalling Technologies (CST), Danvers, MA and included perilipin, peroxisome proliferator-activated receptor gamma (PPARγ; marker for adipogenesis), Runt-related transcription factor 2 (Runx2; marker for osteogenesis), phosphorylated-ACC (P-ACC (Ser79); marker for AMPK activity), phosphorylated AMPKα (P- AMPKα (Thr172); detects active AMPK) and phosphorylated-p70^S6K^ (P-p70^S6K^ (Thr389); upstream regulator of mTOR signalling). The rabbit polyclonal adiponectin antibody was generated in house and the tubulin antibody was bought from Abcam, Cambridge, UK. Antibodies were detected using a LI-COR^®^ Odyssey Infrared Imaging systems and densitometric analysis was carried out using ImageJ software (National Institute of Health, UK) software Version 1.47.

### 2.4 Oil Red O staining

Cells were incubated with adipogenic IID media in the presence or absence of either 10 μM pioglitazone, 500 μM metformin, 100 μM of the AMPK-activator, A769662, or 10 μM of the p70^S6K^-inhibitor, rapamycin. Following differentiation, media was aspirated and cells were fixed to cell culture plates with 10% (v/v) neutral buffered formalin for 30 min. The formalin was then aspirated and staining was carried out with the addition of 0.3% (w/v) Oil Red O in isopropanol:water (60:40) for 5 min in room temperature. The Oil Red O was then aspirated and wells washed with distilled water four times. Imaging was carried out using a Zeiss Axiovert 25 microscope with QImaging camera and supporting software.

### 2.5 AMPK activity assays

AMPK activity was determined in AMPK α1 plus AMPK α2 immuno-complexes through phosphorylation of the peptide HMRSAMSGLHLVKRR [SAMS], as previously described (Morrow et al., 2003). Briefly, the AMPK immunoprecipitates were re-suspended in 20 μl of HEPES Brij-35 buffer. Reaction mixtures (20 μl) containing 5 μl of HEPES Brij-35 buffer, 5 μl of 1 mM SAMS peptide in HEPES Brij-35 buffer, 5 μl of 1 mM AMP in HEPES Brij- 35 buffer and 5 μl of immunoprecipitate re-suspended in HEPES Brij-35 buffer, were prepared in 1.5 ml microcentrifuge tubes on ice and the reaction initiated by the addition of 5 μl of MgATP solution (1 mM [γ-^32^P] ATP, 250–500 c.p.m./pmol; 25 mM MgCl2 in HEPES Brij-35 buffer). Reaction mixtures were then incubated on a vibrating platform in an air incubator at 30 °C for 10 min. Assay mixtures (15 μl) were spotted onto P81 phosphocellulose paper, and rinsed, with gentle stirring to remove free ATP, for 5 min in 1% (v/v) phosphoric acid. A further 2 × 5 min water washes were performed on the phosphocellulose paper, before a final 5 min wash with 1% (v/v) phosphoric acid. A Beckman Multi-Purpose scintillation counter LS 6500 was used to measure [^32^P]-labelled substrate. 3 ml of scintillation fluid was used per sample. Results were corrected for radioactivity recovered in blank reactions lacking the SAMS peptide. One unit of AMPK activity is that required to incorporate 1 nmol of ^32^P into the SAMS substrate peptide/min/mg protein.

### 2.6 Transient transfection and luciferase assay

C3H10T1/2 cells were transfected with the indicated plasmids in 6-well plates, with 1.125 μg/well PPRE (PPARγ reporter, purchased from Adgene) and 6xOSE reporter constructs (Runx2 reporter, supplied by Jian Huang, Rush Medical Centre, Chicago, USA) using Lipofectamine 2000 reagent (Invitrogen, Carlsbad, CA) and then treated with metformin, A769662 or rapamycin with and without adipogenic differentiation media. Cells were then harvested 48 h after transfection and assayed using the Luciferase reporter assay system (Promega, Madison, WI) according to manufacturer's instructions. As a transfection control, the *Renilla* plasmid 0.125 μg/well was co-transfected in each transfection experiment, and the luciferase activity was normalised to the *Renilla* activity.

### 2.7 Statistical analysis

All experiments were performed in triplicate and statistical analysis was performed using Student's t-test or one-way ANOVA. Results are expressed as mean ± standard error (SEM) and differences with *p* < 0.05 were considered statistically significant.

## 3 Results

### 3.1 Metformin suppresses adipogenesis in C3H10T1/2 MSCs

**Fig. 1:**
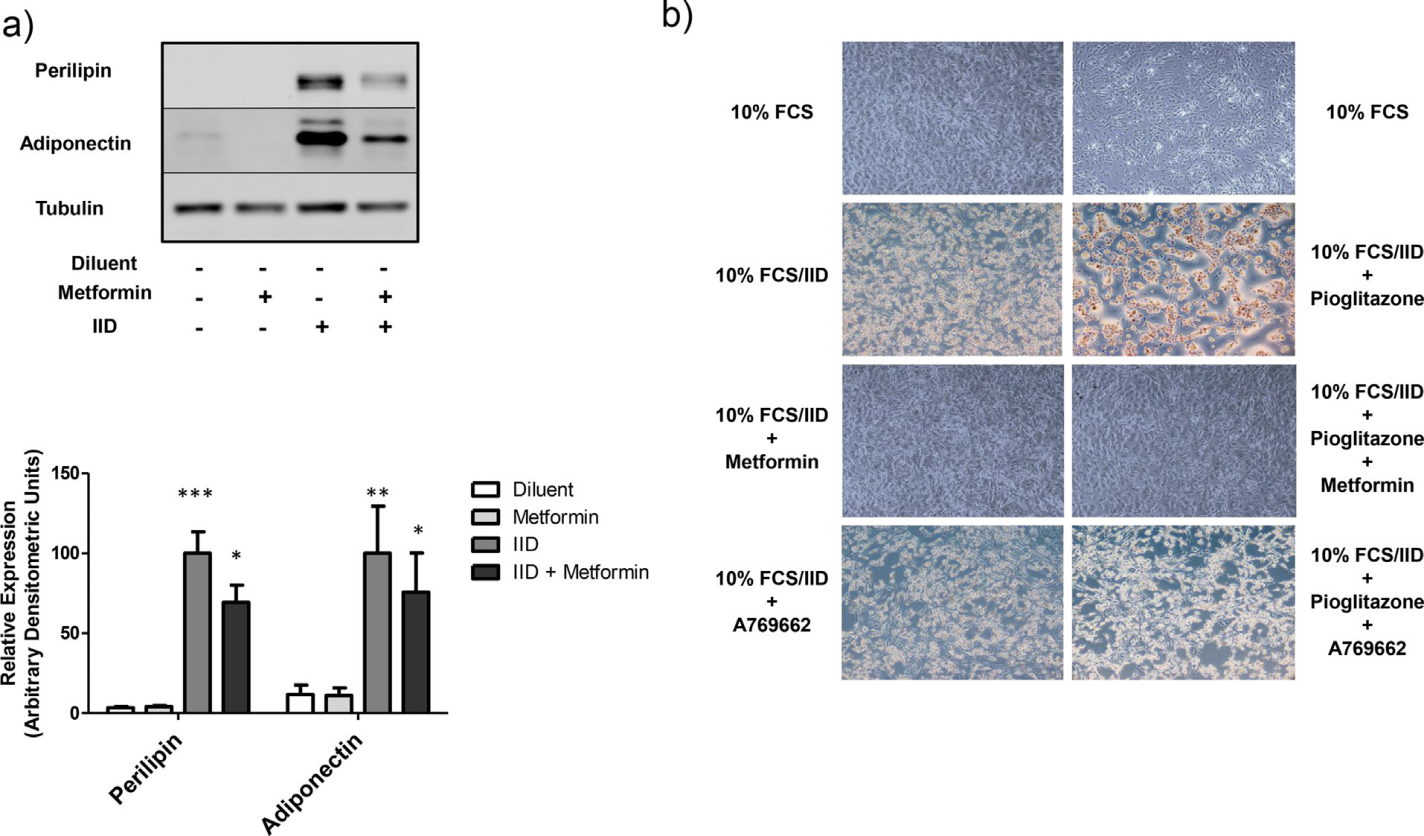
**Metformin and the AMPK-activator, A769662, suppress adipogenesis of CH3H10T1/2 MSCs**. A) Confluent CH3H10T1/2 MSCs were induced to differentiate by addition 10% foetal calf serum (FCS) in the presence or absence of adipogenic IID medium (insulin, isobutylmethylxanthine and dexamethasone) and/or 10 μM pioglitazone (PIO), 500 μM metformin or 100 μM A769662. After 5 days cells were fixed with formalin and stained with Oil Red O to detect neutral lipid accumulation. Representative micrographs from an experiment carried out on three separate occasions with similar results are shown. B) Confluent CH3H10T1/2 MSCs were induced to differentiate by addition 10% FCS in the presence or absence of IID medium and/or 500 μM metformin. Cell extracts were then prepared after 5 days and immunoblotted with antibodies to perilipin, adiponectin and tubulin. Representative immunoblots from an experiment carried out on three separate occasions with similar results are shown. Densitometric analysis of three immunoblots are shown as means ± SEM in the *lower panel*. Significant increases relative to control are indicated, *p < 0.05 and ***, *p* < 0.001.

It has been previously reported that treatment of preadipocyte cell lines with AMPK activators inhibits their conversion to fat cells (Lee et al., 2011, Habinowski and Witters, 2001. Paradoxically, however, the widely used AMPK inhibitor, compound C, has also been reported to inhibit adipogenesis of preadipocyte cell lines (Nam et al., 2008). To try and address this apparent contradiction and to further investigate the role of AMPK in the control of adipogenesis of multipotent mesenchymal stem cells (MSCs), we stimulated murine C3H10T1/2 MSCs with two known activators of AMPK, metformin (500 μM) and A769662 (100 μM). Confluent cultures of C3H10T1/2 MSCs were treated for 5 days with medium containing 10% foetal calf serum (FCS) supplemented with either an insulin-containing, adipogenic medium (IID) and/or the anti-diabetic drug, pioglitazone (PIO), which is a known agonist of the adipogenic transcription factor, PPARγ (Day and Bailey, 2007). Cells were also incubated with 10% FCS alone, as negative control for differentiation. After 5 days of treatment, cells were fixed and then stained with the neutral lipid stain, Oil Red O, to monitor lipid accumulation, which is a widely used late marker of adipogenesis (Fig. 1a). We found that treatment of cells with IID-containing medium stimulated lipid accumulation in C3H10T1/2 MSCs, an effect that was further enhanced by co-treatment of cells with PIO (Fig. 1a). We also found that treatment of cells with either metformin or A769662 suppressed adipogenesis promoted by IID alone or by a combination of IID plus PIO (Fig. 1A), with metformin being a more effective inhibitor of lipid accumulation (Fig. 1A). The effects of metformin on the suppression of adipogensis was confirmed by western blotting for two late markers of fat cell conversion, adiponectin and perilipin, the expression of which were strongly induced following IID treatment, but were suppressed in the presence of metformin (Fig. 1B).

**Fig. 2:**
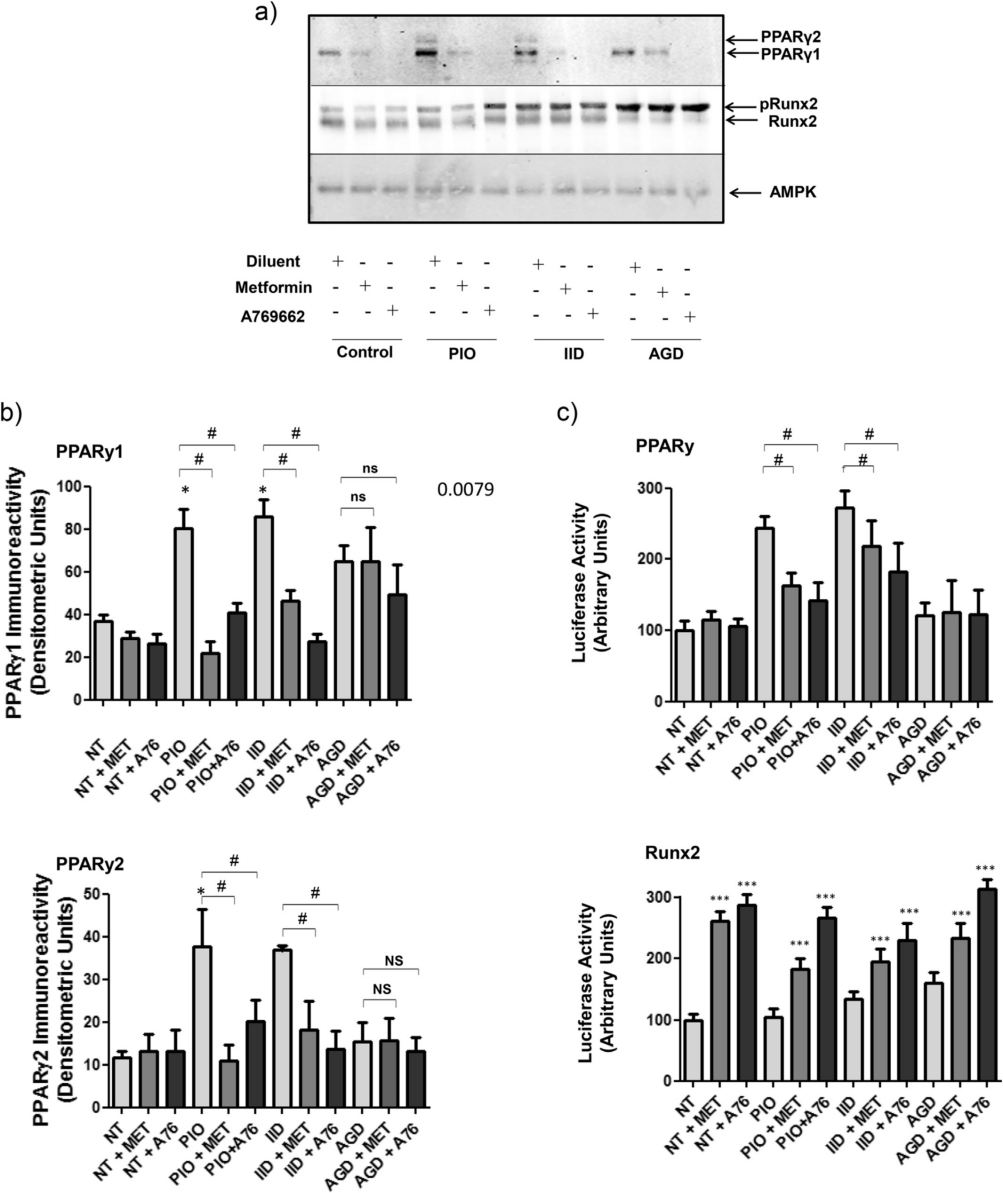
**Effects of Metformin and the AMPK-activator, A769662, on early markers of differentiation in C3H10T1/2 cells**. A) Confluent CH3H10T1/2 cells were stimulated for 5 days with 10 μM pioglitazone (PIO) or an adipogenic (IID) or osteogenic (AGD) cocktail, in the presence or absence of 500 μM metformin or 100 μM A769662. Cell extracts were then prepared and immunoblotted with antibodies to PPARγ, Runx2 and AMPK. The phosphorylation-dependent electrophoretic mobility shift of Runx2 induced by AGD treatment is indicated (pRunx2). Representative immunoblots from an experiment carried out on three separate occasions with similar results are shown. B) Densitometric analysis of PPARγ1 (*upper panel*) and PPARγ2 (*lower panel*) levels relative to Runx2 are shown as means ± SEM. Significant increases (*, *p* < 0.05) relative to control, and significant decreases relative to PIO-stimulated cells (#, *p* < 0.05), are indicated (n = 3). Non-significant changes are also indicated (ns). C) Confluent C3H10T1/2 cells were transfected with a PPARγ (*upper panel*) and Runx2 (*lower panel*) luciferase gene reporter constructs, together with control *Renilla* luciferase vector, and then stimulated for two days with 500 μM metformin or 100 μM A769662, in the presence or absence of 10 μM pioglitazone (PIO), adipogenic medium (IID) or osteogenic medium (AGD). Cell extracts were then prepared and luciferase activities were measured using a dual luciferase reporter assay. Luciferase activities from three separate experiments are shown as means ± SEM. Significant increases in luciferase activity are indicated; *, *p* < 0.05, **, *p* < 0.01 and ***, *p* < 0.001, as are significant decreases in activity, #, *p* < 0.05 (n = 3). Non significance is also indicated (ns).

These results suggest that AMPK activators inhibit adipogenesis of C3H10T1/2 MSCs. To further elucidate the mechanisms of action of metformin and A769662 in these cells, we next treated cells with 10% FCS, supplemented with either IID or PIO, and measured the protein expression levels of the transcription factor, PPARγ, which is a widely used early marker of adipogenesis (Fig. 2A and B). To complement these experiments, we also measured the activation of PPARγ transcriptional activity, by transfecting C3H10T1/2 cells with PPARγ-responsive luciferase reporter construct (Fig. 2C). We found that treatment of cells for 5 days with 10% FCS in the presence of either IID or PIO, induced a significant increase in the two PPARγ splice variants, PPARγ1 and PPARγ2 (Fig. 2A and B), which correlated with a significant increase in PPARγ transcriptional activity, as determined by luciferase assay (Fig. 2c). In agreement with the Oil Red O staining experiments in Fig. 1, we found that treatment of cells with either metformin or A769662 effectively suppressed IID- and PIO-stimulated increases in PPARγ protein levels (Fig. 2A and B), as well as IID- and PIO-stimulated PPARγ activity (Fig. 2C), as determined by gene reporter assays. Given that increases in PPARγ activity during the early stages of adipogenesis are necessary and sufficient to promote terminal fat cell development (Rosen and Spiegelman, 2000), it appears that the ability of metformin and A769662 to inhibit adipogenesis of C3H10T1/2 MSCs is linked to their ability to suppresses increases in PPARγ protein levels promoted by treatment of cells with either IID or PIO. Moreover, given that the PPARγ luciferase reporter assays were carried out after only two days of differentiation, it appears that the suppressive actions of metformin and A769662 occur at a very early stage of the adipogenic process.

The control of differentiation of MSCs into fat and bone is thought to be controlled through reciprocal regulation of PPARγ and the osteogenic transcription factor, Runx2 (Jeon et al., 2003, Muruganandan et al., 2009), during the commitment stage of differentiation. We therefore also examined Runx2 protein levels (Fig. 2A and B) and activity (Fig. 2C). As a positive control for these experiments, cells were incubated with 10% FCS supplemented with a widely used osteogenic medium (AGD) (Shea et al., 2003) to induce Runx2 activity. We found that treatment of cells with 10% FCS plus AGD for 5 days did not significantly affect PPARγ protein levels (Fig. 2A and B) or activity (Fig. 2C). However, AGD treatment alone did promote a noticeable phosphorylation band-shift of Runx2 protein in treated cells (Fig. 2A), which correlated with an increase in Runx2 activity, as determined by a Runx2 gene reporter assay (Fig. 2C). Treatment with either metformin or A769662 significantly increased Runx2 activity in C3H10T1/2 cells (Fig. 2C), which did not correlate with an increase in AGD-promoted phospho-Runx2 levels, as determined by band-shift (Fig. 2A). Since osteogenesis and adipogenesis of MSCs are thought to be reciprocally regulated by the PPARγ:Runx2 activation ratio (Chen et al., 2016), we can conclude that the inhibitory actions of metformin and A769662 on the adipogenesis of C3H10T1/2 MSCs can partly be explained by reciprocal control of PPARγ and Runx2 activity, thereby favouring an osteogenic lineage. In addition, whereby the actions of metformin and A769662 appear to be through the suppression of adipogenic-dependent increases in PPARγ expression, the actions on Runx2 activity remain to be determined, but appear to be independent of osteogenic-linked increases in Runx2 phosphorylation (Fig. 2A).

### 3.2 Metformin suppresses adipogenesis in C3H10T1/2 MSCs through the inhibition of the p70^S6K^ signalling pathway and not through the activation of AMPK

**Fig. 3:**
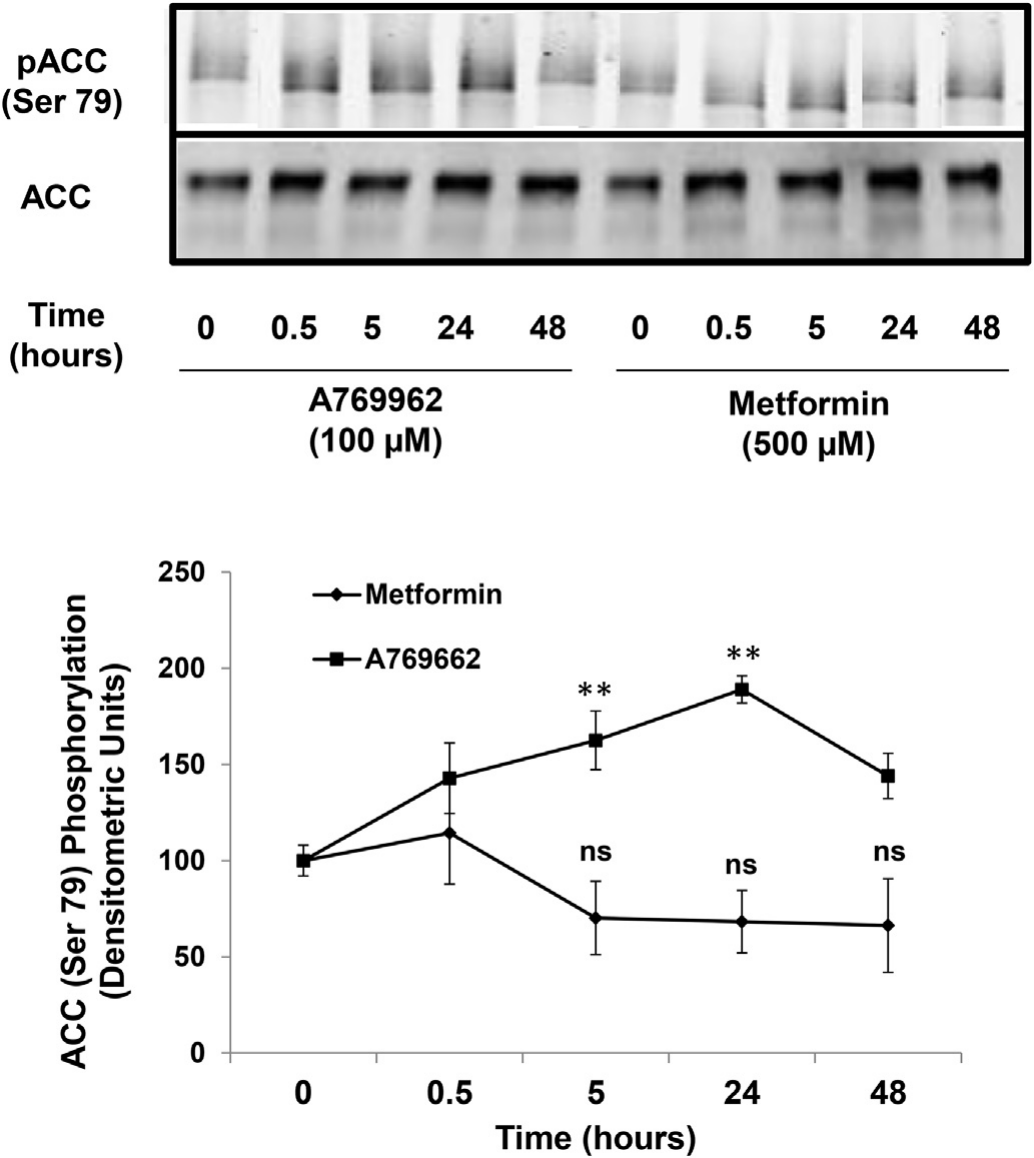
**The AMPK activator, A769662, but not metformin activates AMPK in C3H10T1/2 cells**. Confluent C3H10T1/2 cells were stimulated for the indicated times with 100 μM A769662 or 500 μM metformin. Cell extracts were then prepared and immunoblotted with antibodies towards the phosphorylated form of the AMPK substrate, ACC, or total ACC, as indicated in the *upper panel*. Densitometric values were obtained from immunoblots from three separate experiments and are shown as means ± SEM in the line graph in the *lower panel*. Significant increases in pACC are indicated; **, p < 0.01 (n = 3). Non significance is also indicated (ns).

**Fig. 4:**
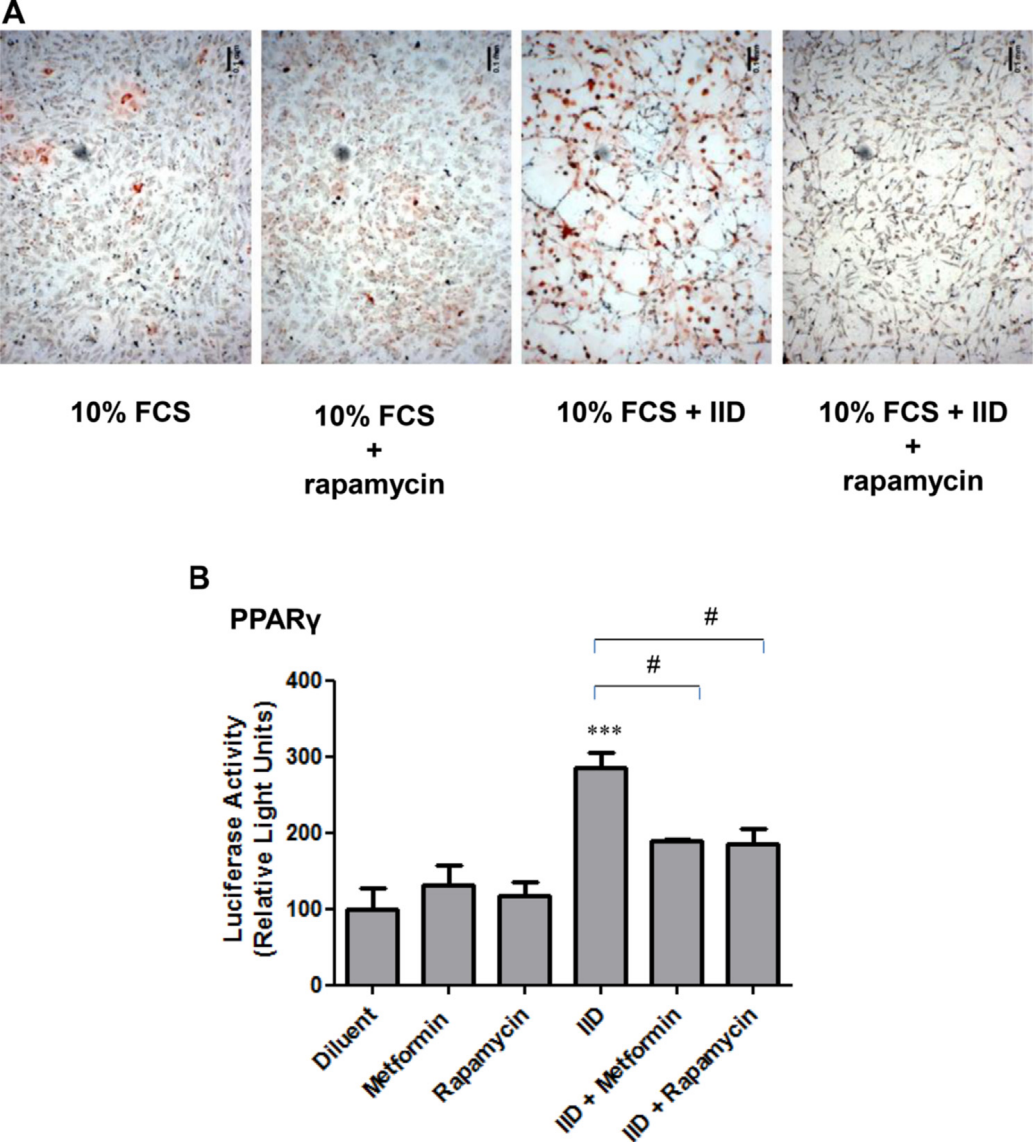
**The mTOR inhibitor, rapamycin, suppresses adipogenesis of C3H10T1/2 cells**. A) Confluent CH3H10T1/2 cells were induced to differentiate by addition of 10% foetal calf serum (FCS) supplemented with adipogenic cocktail (IID), in the presence or absence of the mTOR inhibitor, 10 μM rapamycin. After 5 days cells were fixed with formalin and stained with Oil Red O to detect neutral lipid accumulation. Representative micrographs from an experiment carried out on three separate occasions with similar results are shown. B) Confluent C3H10T1/2 cells were transfected with PPARγ luciferase gene reporter construct, together with control *Renilla* luciferase vector and then stimulated for two days with 500 μM metformin or 10 μM rapamycin, in the presence or absence of IID. Cell extracts were then prepared and luciferase activity was measured using a dual luciferase reporter assay. Luciferase activities from three separate experiments are shown as means ± SEM. Significant increases in PPARγ activity are indicated ***, *p* < 0.001, as are significant decreases in PPARγ activity, #, *p* < 0.05, relative to IID-stimulated cells (n = 3).

**Fig. 5:**
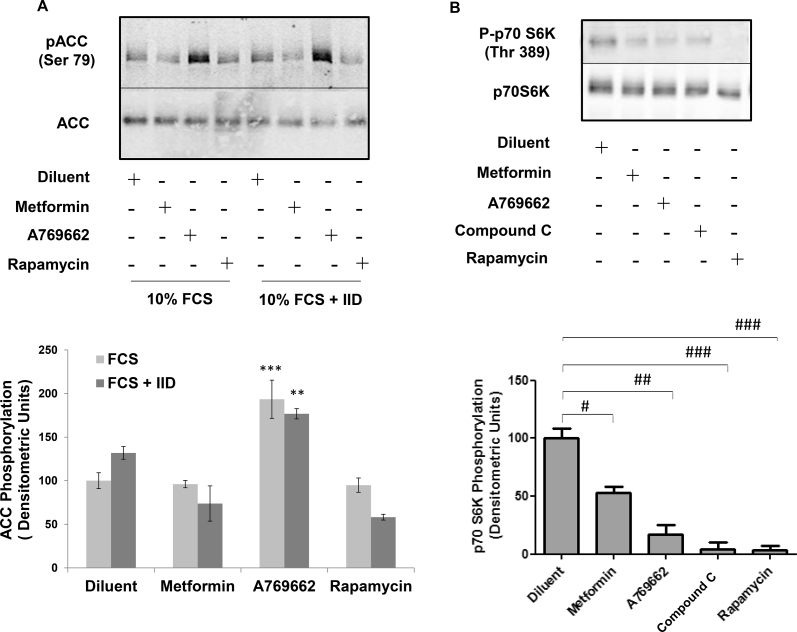
**Effects of rapamycin on AMPK and p70**^**S6K**^**activities in C3H10T1/2 cells**. A) Confluent CH3H10T1/2 cells were stimulated for 5 days with IID, in the presence or absence of 500 μM metformin, 100 μM A769662, 10 μM rapamycin or the AMPK inhibitor, 10 μM compound C. Cell extracts were then prepared and immunoblotted with antibodies to phosphorylated ACC (Ser 79). Representative immunoblots from an experiment carried out on three separate occasions with similar results are shown (*upper panel*). Densitometric values from 3 separate experiments are shown in the *lower panel* as means ± SEM. Significant increases relative to control are indicated, **, *p* < 0.01 and ***, *p* < 0.001 (n = 3). B) Confluent CH310T1/2 cells were stimulated for 5 days with IID, in the presence or absence of 500 μM metformin, 100 μM A769662, 10 μM compound C or 10 μM rapamycin. Cell extracts were then prepared and immunoblotted with antibodies to phosphorylated p70^S6K^ and total p70^S6K^. Representative immunoblots from an experiment carried out on three separate occasions with similar results are shown in the *upper panel*. Densitometric analysis of mean ± SEM p70^S6K^ phosphorylation from 3 separate experiments are shown in the *lower panel*. Significant increases (*, *p* < 0.05) relative to control, and significant decreases relative to IID-stimulated cells (##, *p* < 0.01 and ###, *p* < 0.001), are indicated (n = 3).

Both metformin and A769662 are reported to activate AMPK in a variety of cell types (Zhou et al., 2001, Cool et al., 2006). Given the inhibitory effects of these two compounds on early and late markers of adipogenesis of C3H10T1/2 MSCs (Fig. 1, Fig. 2), we next tested their ability to activate AMPK in these cells. We did this by measuring the phosphorylation of a known AMPK substrate, Ser 79 of acetyl coenzyme carboxylase (ACC), using phospho-specific antibodies. Intriguingly, although A769662 provoked a robust and rapid phosphorylation of ACC, which was maintained for up to 48 h, metformin did not induce a significant phosphorylation of ACC, even after 48 h stimulation (Fig. 3). It is likely, therefore, that while metformin is an effective inhibitor of the adipogenic differentiation of C3H10T1/2 MSCs, in response to insulin-containing IID medium, or activation of PPARγ by PIO, this occurs through mechanisms that are independent of AMPK activation. Recent work, however, has shown that the control of osteogenesis is regulated through interactions between PPARγ and the mTOR/p70^S6K^ signalling pathway (Sun et al., 2013). Moreover, metformin has been shown to inhibit the activation of the p70^S6K^ pathway independently of AMPK (Vazquez-Martin et al., 2009) in tumour cells and p70^S6K^ has been shown to be required for the growth hormone-dependent adipose conversion of 3T3-F442A preadipocytes (Yarwood et al., 1999). We therefore examined the role of the p70^S6K^ pathway on IID-induced adipogenesis of C3H10T1/2 cells by incubating cells with the mTOR/p70^S6K^ inhibitor, rapamycin. We found that incubation of differentiating cells with rapamycin dramatically inhibited lipid accumulation associated with adipogenesis, as determined by Oil Red O staining (Fig. 4A). Furthermore, rapamycin also suppressed PPARγ activity, as determined by gene reporter assays, indicating that the mTOR/p70^S6K^ pathway is required for adipogenesis of C3H10T1/2 cells (Fig. 4B). We found that the effects of rapamycin were specific to inhibition of mTOR/p70^S6K^, since rapamycin treatment had no significant effect of phospho-ACC levels (Fig. 5A), indicating no effect on AMPK activity, but, rather, significantly inhibited phosphorylation of p70^S6K^ on Thr 389 (Fig. 5B), which is the mTOR phosphorylation site critical for kinase function (Saitoh et al., 2002). Importantly, both metformin and A769662 also inhibited p70^S6K^ phosphorylation (Fig. 5B), indicating that suppression of adipogenesis of C3H10T1/2 MSCs by these compounds may involve suppression of mTOR/p70^S6K^ signalling at early stages of commitment to differentiation.

**Fig. 6:**
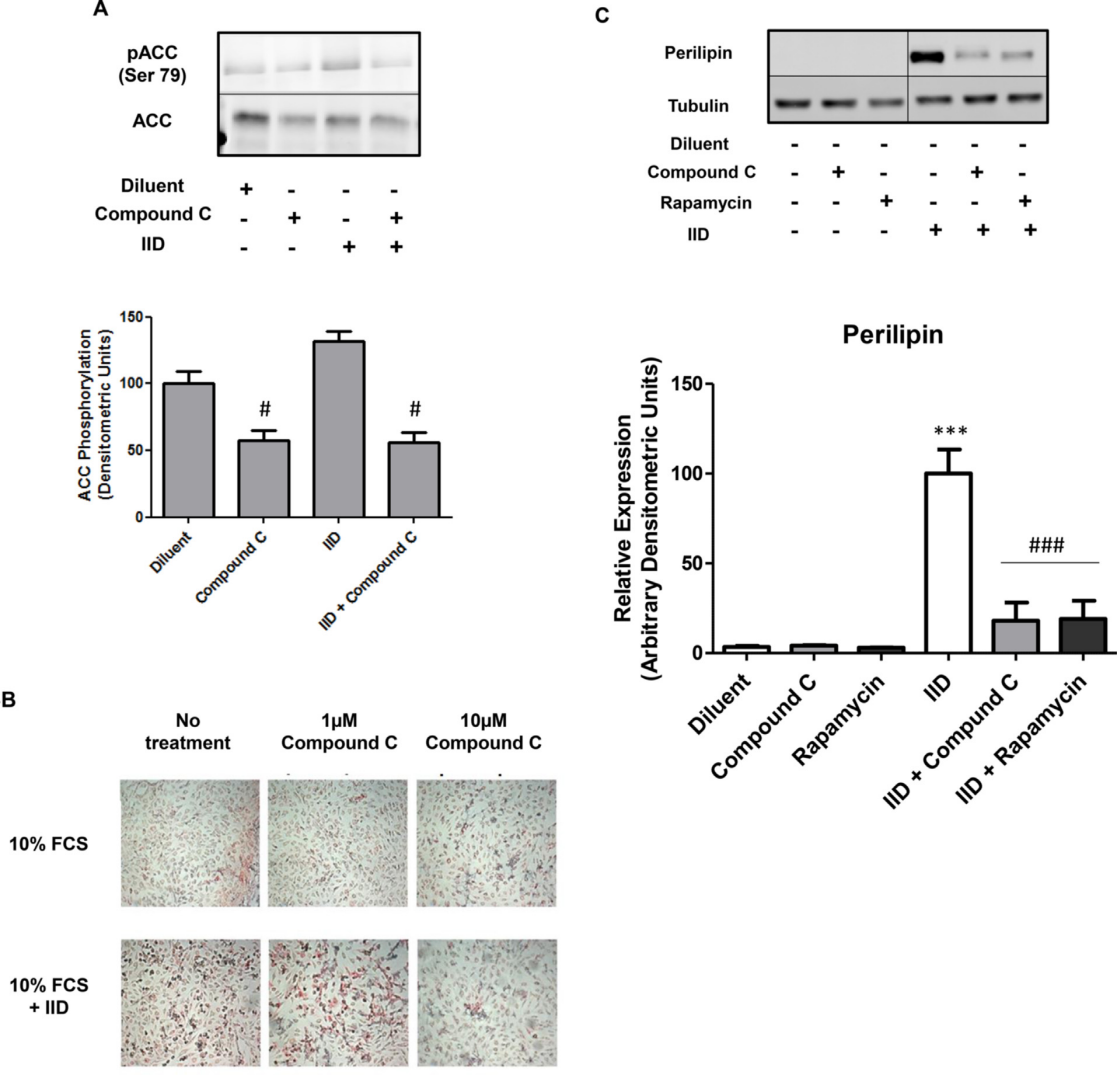
**Compound C inhibits adipogenesis of C3H10T1/2 cells**. A) Confluent CH3H10T1/2 cells were stimulated for 5 days with IID, in the presence or absence of the AMPK inhibitor, 10 μM compound C. Cell extracts were then prepared and immunoblotted with antibodies to phosphorylated ACC (Ser 79). Representative immunoblots from an experiment carried out on three separate occasions with similar results are shown (*upper panel*). Densitometric values from 3 separate experiments are shown in the *lower panel* as means ± SEM. Significant decreases relative to control are indicated, #, p < 0.05. B) Confluent CH3H10T1/2 cells were treated with 10% FCS supplemented with adipogenic cocktail (IID), in the presence or absence of the indicated concentrations of compound C. After 5 days cells were fixed with formalin and stained with Oil Red O to detect neutral lipid accumulation. Representative micrographs from an experiment carried out on three separate occasions with similar results are shown. C) Confluent CH3H10T1/2 MSCs were induced to differentiate by addition 10% FCS in the presence or absence of IID medium and/or 10 μM Compound C or 10 μM rapamycin. Cell extracts were then prepared after 5 days and immunoblotted with antibodies to perilipin and tubulin. Representative immunoblots from an experiment carried out on three separate occasions with similar results are shown. Densitometric analysis of three immunoblots are shown as means ± SEM in the *lower panel*. Significant increases relative to control are indicated, ***p < 0.001 and significant decreases with respect to IID-treated cells are indicated, ###, *p* < 0.001.

Although we found that metformin inhibits mTOR/p70^S6K^ signalling apparently independently of AMPK activation (Fig. 3, Fig. 5A), this does not rule out a role for AMPK in the control of p70^S6K^ activation. In fact, it has been reported that AMPK inhibits mTOR/p70^S6K^ signalling, which is thought to underlie the actions of metformin in a range of cellular contexts (Viollet et al., 2012, Dowling et al., 2011). To determine whether the same relationship exists in C3H10T1/2 MSCs, we incubated cells with the AMPK inhibitor, compound C, and determined its action on ACC (Ser 79) and p70^S6K^ (Thr 389) phosphorylation (Fig. 5A and B, respectively). We found that compound C significantly inhibited both basal p70^S6K^ (Thr 389; Fig. 5B) and ACC (Ser 79; Fig. 6A) phosphorylation, suggesting that AMPK is linked to the activation of mTOR/p70^S6K^ signalling in these cells. We also found that 10 μM compound C was able to inhibit adipogenesis of C3H10T1/2 cells treated with IID, as determined by lipid accumulation (Fig. 6B) and expression of the adipogeneic marker, perilipin (Fig. 6C), as well as suppressing AMPK activation in the presence or absence of IID (Fig. 6A). This suggests that basal levels of AMPK activity, perhaps acting through the mTOR/p70^S6K^ pathway (Fig. 5B), are important for supporting adipose conversion of these cells.

### 3.3 AMPK plays a dual role in regulating the adipogenesis of mouse embryonal fibroblasts (MEFs)

**Fig. 7:**
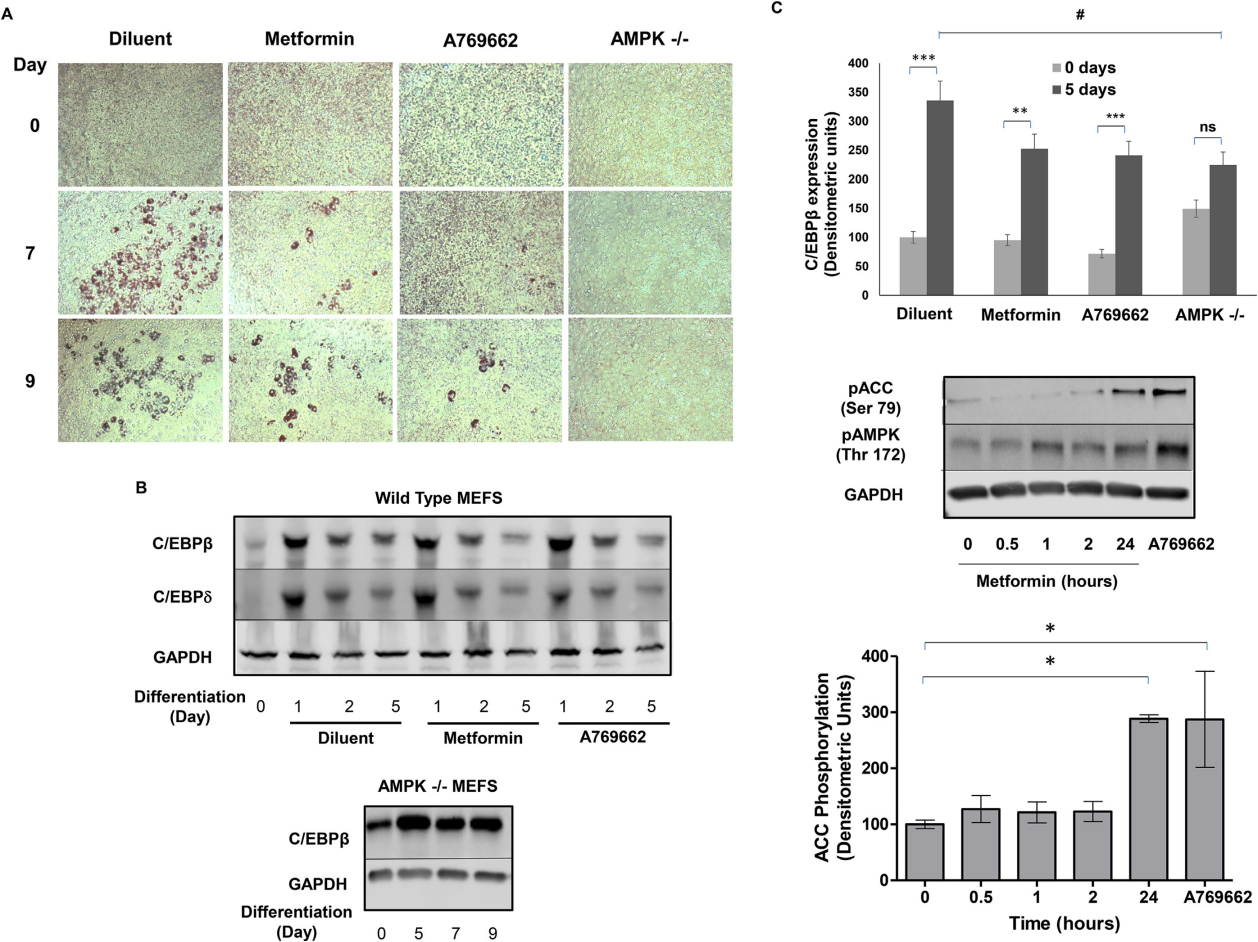
**Metformin suppresses adipogenesis in MEFs**. A) Wild type and AMPK knockout (−/−) mouse embryonic fibroblasts (MEFs) were treated with IID plus 10 μM pioglitazone (PIO) for 7 or 9 days as indicated. Wild type MEFs were also stimulated in the presence or absence of 1 mM metformin or 100 μM A769662. Cells were then stained with Oil Red O. Representative micrographs from an experiment carried out on three separate occasions with similar results are shown. B) Confluent wild-type (*upper panel*) and AMPK−/− (*lower panel*) MEFs were stimulated for the indicated times with 10 μM pioglitazone (PIO) plus (IID) and, for wild-type MEFs, in the presence or absence of 1 mM metformin or 100 μM A769662. Cell extracts were then prepared and immunoblotted with antibodies to C/EBPβ and/or C/EBPδ, as indicated. Representative immunoblots from experiments carried out on three separate occasions with similar results are shown. C) Densitometric values taken at day 5 from the experiment carried out in Fig. 7B are shown in the *lower panel* as means ± SEM. Significant increases in expression are indicated, **, *p* < 0.01 and ***, *p* < 0.001. Significant decreases relative to control are also indicated, #, *p* < 0.05. Non-significance is also indicated (ns). D) Wild type MEFs were incubated with 1 mM metformin for the times indicated or with 100 μM A769662 for 30 min. Cells were then lysed and the lysates were immunoblotted with antibodies specific to the indicated proteins (*upper panel*). Results from densitometric analysis of three separate immunoblots are shown in the *lower panel* as means ± SEM. Significant increases relative to t = 0 are indicated, *, *p* < 0.05.

Our findings in C3H10T1/2 cells may provide the explanation for the apparently conflicting, previous reports that both activation AMPK and inhibition of AMPK block adipogenesis of 3T3-L1 preadipocytes (Lee et al., 2011, Habinowski and Witters, 2001, Nam et al., 2008). This has been suggested to be a result of AMPK exerting differential control during the process of differentiation; due to AMPK exerting different control at early time points versus late time points. Instead, we hypothesise there is a threshold level of AMPK activity required for adipogenesis, above which further activation leads to a break on the process. Indeed, it is worth noting that AMPK activity levels do not change significantly during adipogeneis of 3T3-L1 preadipocytes (Supplementary Fig. 1) and C3H10T1/2 MSCs (unpublished observations) indicating that AMPK activity must be kept under stringent control to allow the differentiation of these cells. To investigate this relationship further, we next examined the effects of metformin and A769662 and adipogenesis in wild type and AMPKα knockout (−/−) mouse embryonal fibroblasts (MEFs). We first incubated wild type or AMPKα (−/−) MEFs with 10% FCS, in the presence or absence of a combination of adipogenic medium (IID) plus PIO, (Fig. 7A). Cells were then fixed and stained with Oil Red O at days 7 and 9, to monitor late-stage triglyceride accumulation (Fig. 7A), and cell extracts were prepared at days 1, 2 and 5, to detect levels of the early marker of differentiation, the transcription factor C/EBPβ, by western blotting (Fig. 7B). We found that a combination of IID and PIO promoted a large increase in lipid accumulation in wild type MEFs but not AMPKα (−/−) MEFs, at days 7 and 9 (Fig. 7A). We also found that after an initial increase in the expression of C/EBPβ and C/EBPδ at day 1 in wild type MEFs, following IID and PIO treatment, levels fell by day 5 but remained significantly above basal (Fig. 7B). This was not the case in AMPKα (−/−) MEFs, where C/EBPβ levels returned to basal by day 5 and remained at that level for up to 9 days of treatment with differentiation medium (Fig. 7B). These results suggest that a basal level of AMPKα is required for adipogenesis of MEFs and exerts actions on both early and late markers of adipose conversion. We next examine the effects of metformin and A769662 on adipogenesis of MEFs in response to IID and PIO treatment. We found that treatment of wild type MEFs with either metformin or A769662 led to a reduction in lipid accumulation (Fig. 7A) and C/EBPβ protein levels (Fig. 7B), indicating that both compounds are anti-adipogenic in these cells. We also found that both metformin (1 mM) and A769662 (100 μM) significantly increased phosphorylation of ACC at (Ser 79; Fig. 7C), indicating that they both exert their anti-adipogenic actions through the activation of AMPK. Together with the results obtained from CH310T1/2 MSCs this suggests that the role of AMPK in the control of adipogensis is complex, suggesting both positive and negative regulation that may depend on the cellular context. Moreover, the anti-adipogenic actions of the anti-diabetic drug, metformin, may be both AMPK-dependent and AMPK-independent, again depending on the cellular context.

## 4 Discussion

Previous work has shown that AMPK activation reduces adipogenesis in favour of osteogenesis in adipocyte-derived human MSCs (hMSCs) and bone marrow-derived MSCs (Kim et al., 2012, Lee et al., 2014). In the present work, we found that the AMPK activators, metformin and A769662, inhibited adipogenesis in murine C3H10T1/2 MSCs and in wild type MEFs. Both AMPK activators promoted a significant activation of AMPK in wild type MEFs, although, interestingly, we found that metformin, did not promote AMPK activation in C3H10T1/2 cells (Fig. 3). Metformin has been shown to activate AMPK in many different cell types; however a requirement of AMPK for the therapeutic actions of metformin has been questioned following genetic loss of function experiments that demonstrated AMPK-independent mechanisms of action of metformin during the inhibition of hepatic gluconeogenesis (Foretz et al., 2010). The AMPK-independent action of metformin on the inhibition of adipogenesis reported here might be due to cell type specific effects or stage-specific effects during the differentiation process. For example, most of the studies demonstrating an AMPK-dependent action of metformin were conducted in more differentiated cell lines, such as pre-osteoblasts (Jang et al., 2011, Kanazawa et al., 2008, Cortizo et al., 2006), pre-adipocytes (Moreno-Navarrete et al., 2011, Lee et al., 2012), myoblasts (Longnus et al., 2005, Kobashigawa et al., 2014, Fulco et al., 2008) and neuronal mouse cell lines (Bang et al., 2014), instead of the more primitive cell progenitors investigated here.

With regards to stage-specific effects, Pantovic et al. (Pantovic et al., 2013) demonstrated that there is a coordinated time-dependent activation of different signalling pathways during the osteogenic differentiation of hMSCs, which is AMPK-dependent in the early stages of differentiation followed by late stage activation of the Akt/mTOR signalling pathway. Given the results presented here, it could be argued that similar mechanisms might regulate adipogenesis in murine MSCs. For example, we find an overall requirement for basal levels of AMPK activity for adipogenesis of C3H10T1/2 cells, as demonstrated by the use of the AMPK inhibitor compound C (Fig. 6B) and verified by the use of AMPK knockout (−/−) MEFs (Fig. 7A). It should be noted, however, that AMPK activity levels remain constant throughout the process of adipogenesis, as determined by AMPK activation assays (Supplementary Fig. 1) and phosphorylation of ACC on Ser 79 (results not shown). Despite this, the ability of metformin to inhibit adipogenesis was found to be due to a reduction in the PPARγ:Runx2 activation ratio (Fig. 2C) and this was linked to the inhibition of mTOR/p70^S6K^ signalling (Fig. 4). This suggests that the ability of metformin to control the commitment of MSCs to differentiate into either osteoblasts or adipocytes is governed at an early stage through the inhibition of mTOR/p70^S6K^ signalling. Moreover, wild type MEFs were observed to accumulate lipid and increase expression of C/EBPβ in response to an adipogenic cocktail of IID plus PIO (Fig. 7). These effects were blocked in AMPK (−/−) MEFs, which may indicate that AMPK is required for efficient, late stage lipid accumulation or, since AMPK is involved in mitochondrial biogenesis, altered mitochondrial function (Bergeron et al., 2001).

Overall, our work suggests that metformin exerts multiple effects to inhibit adipogenesis in different cell types. Therefore, the overriding view that metformin exerts its effects on adipogenesis simply by promoting AMPK activation may therefore need some revision. In particular, the role of AMPK itself appears to be complex, in that it appears to exert both positive and negative effects during the adipogenic conversion of MEFs and C3H10T1/2 MSCs. In conclusion, further investigation into how metformin suppresses signalling through the mTOR/p70^S6K^ pathway may lead to new therapeutic intervention strategies to prevent unwanted bone marrow adipogenesis associated with diseases, such as T2DM, where bone health is impaired.

## Appendix A Supplementary data

Supplementary Fig. 1 **AMPK Activity remains constant throughout adipogenesis**. Cell lysates were prepared from 3T3-L1 preadipocytes at the indicated time points throughout adipogenesis. Anti-α1 and anti-α2 antibodies were used to immunoprecipitate total AMPK complexes from 3T3-L1 cell extracts (100 μg). Immunoprecipitates were then assayed for AMPK activity as described in Materials and methods. Data shown represents the mean ± S.E.M from three independent experiments. Supplementary Fig. 1
