# Corrigendum to “Metformin suppresses adipogenesis through both AMP-activated protein kinase (AMPK)-dependent and AMPK-independent mechanisms” [Mol. Cell. Endocrinol. 440 15 January 2017 57–68]

**DOI:** 10.1016/j.mce.2017.01.049

**Published:** 2017-03-05

**Authors:** S.C. Chen, R. Brooks, J. Houskeeper, S.K. Bremner, J. Dunlop, B. Viollet, P.J. Logan, I.P. Salt, S.F. Ahmed, S.J. Yarwood

**Affiliations:** a The Developmental Endocrinology Research Group, School of Medicine, University of Glasgow, Glasgow G51 4TF, UK; b Institute of Molecular, Cell and Systems Biology, University Avenue, University of Glasgow, Glasgow G12 8QQ, UK; c INSERM, U1016, Institut Cochin, Paris, France; d CNRS, UMR8104, Paris, France; e Université Paris Descartes, Sorbonne Paris Cité, France; f Institute of Cardiovascular and Medical Sciences, University Avenue, University of Glasgow, Glasgow G12 8QQ, UK; g Institute of Biological Chemistry, Biophysics and Bioengineering, Edinburgh Campus, Heriot-Watt University, Edinburgh EH14 4AS, UK

The authors regret that the crop lines in the western blot in Figure 6c were put in the wrong place when the figure was prepared. The corrected version of the figure is:

**Figure:**
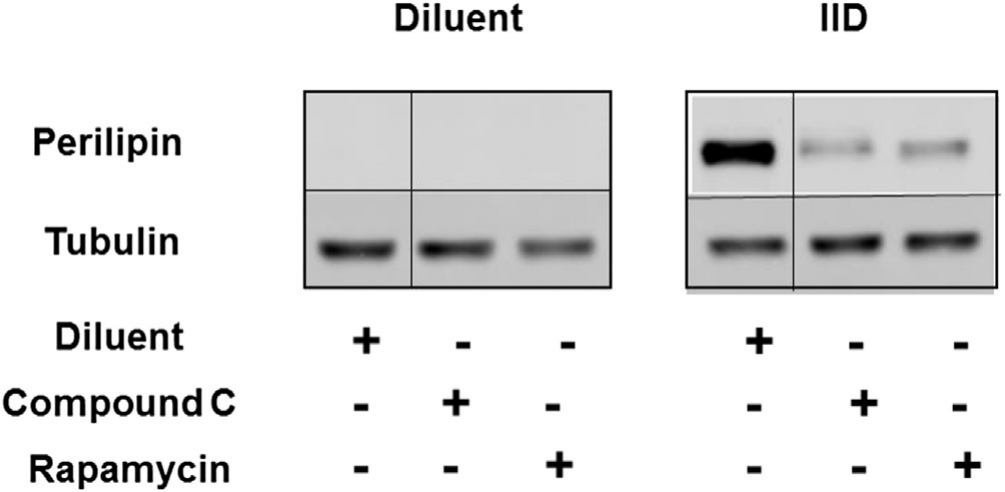

Revising the figure does not alter the densitometry measurements we made or change the conclusions we draw from the data. The authors would like to apologise for any inconvenience caused.

