# Supplementary figures and images for: Metformin suppresses adipogenesis through both AMP-activated protein kinase (AMPK)-dependent and AMPK-independent mechanisms
Source: Mol Cell Endocrinol. 2017 Jan 15;440:57–68. doi: 10.1016/j.mce.2016.11.011 (PMC5228588; doi:10.1016/j.mce.2016.11.011)

Supplementary Figure 1

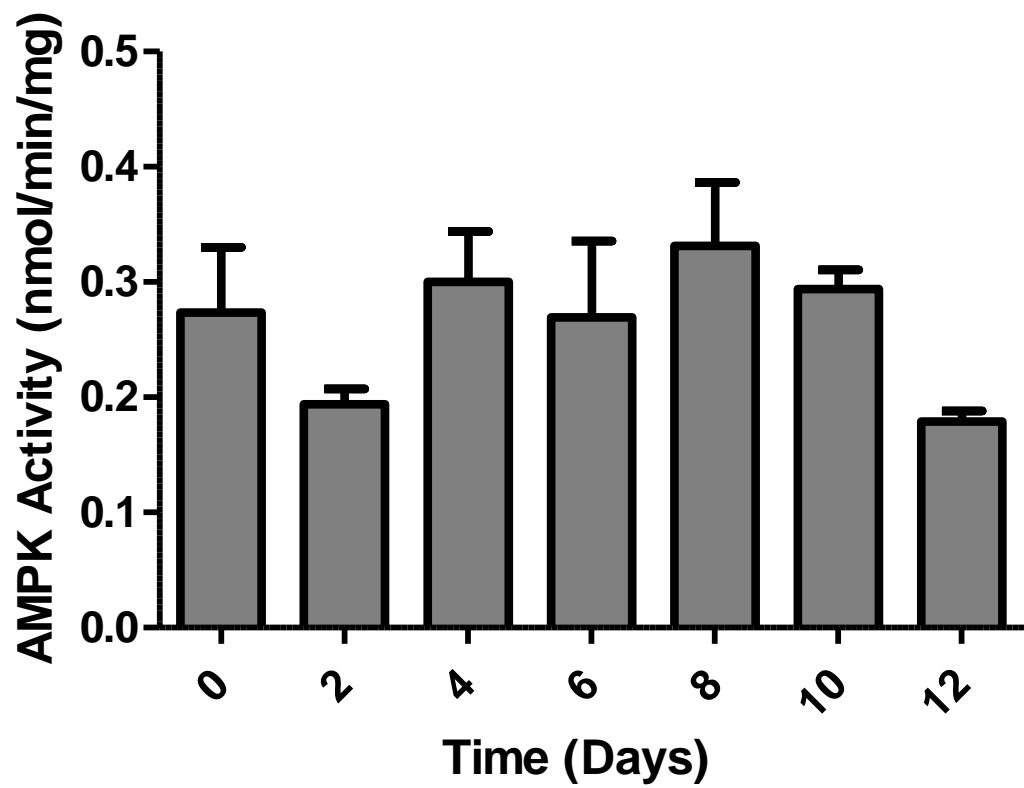

Supplement: Supplementary Fig. 1 — AMPK Activity remains constant throughout adipogenesis. Cell lysates were prepared from 3T3-L1 preadipocytes at the indicated time points throughout adipogenesis. Anti-α1 and anti-α2 antibodies were used to immunoprecipitate total AMPK complexes from 3T3-L1 cell extracts (100 μg). Immunoprecipitates were then assayed for AMPK activity as described in Materials and methods. Data shown represents the mean ± S.E.M from three independent experiments. [file mmc1.pdf]
